# Atopic keratoconjunctivitis complicated by *Kocuria koreensis* keratitis: the first case

**DOI:** 10.1186/s13223-017-0178-9

**Published:** 2017-01-25

**Authors:** Noriko Inada, Jun Shoji, Satoru Yamagami

**Affiliations:** Department of Visual Sciences, Division of Ophthalmology, School of Medicine, Nihon University, 30-1, Oyaguchi-Kamicho, Itabashi-ku, Tokyo 173-8610 Japan

**Keywords:** Atopic dermatitis, Atopic keratoconjunctivitis, *Kocuria* keratitis, Shield ulcer

## Abstract

**Background:**

Patients with atopic dermatitis have a predisposition to *Staphylococcus aureus* and a *Herpes simplex* virus infection. The treatment of atopic diseases with steroid and immunosuppressive agents induces opportunistic infection. However, there is a concern regarding visual prognosis in patients with atopic keratoconjunctivitis (AKC) complicated with infectious keratitis. We report an unusual case of an atopic shield ulcer with *Kocuria* keratitis.

**Case presentation:**

A 51-year-old Japanese man presented with a 14-day history of eye pain and visual loss in his left eye. At the initial examination, a shield ulcer was observed in the upper-central cornea of the left eye, and the conjunctiva in both eyes had a velvety appearance due to papillary formation, as well as hyperemia and swelling in the palpebral area. The shield ulcer showed white stromal opacification in the marginal zone with a coral-like appearance. Samples were obtained by corneal scraping, and *Kocuria* sp. was identified by microbiological examination including culture and matrix-assisted laser desorption/ionization time-of-flight mass spectrometry. The 16S rRNA gene sequence analysis was performed using isolated Kocuria strain from the patient. The obtained DNA sequence showed 99% homology with *Kocuria koreensis.* The combination of corneal scraping and instillation of cefmenoxime antibiotic ophthalmic solution was considered useful for the treatment of *Kocuria* keratitis.

**Conclusion:**

Clinicians should be aware of *Kocuria* keratitis as a corneal complication of AKC, and that rapid diagnosis of *Kocuria* keratitis may improve visual prognosis.

## Background

Atopic keratoconjunctivitis (AKC) is a severe chronic allergic conjunctival disease often associated with atopic facial dermatitis. Corneal complications of AKC, including keratoconus, shield ulcer, corneal opacity with neovascularization, and infectious keratitis, impact significantly on the visual prognosis of AKC [1].

Patients with atopic dermatitis are predisposed to *Staphylococcus aureus* and *Herpes simplex* virus skin infections, with potential resultant complications such as contagious impetigo and Kaposi’s varicelliform eruption. Similarly, AKC may be complicated by infectious keratitis such as staphylococcal keratitis and *Herpes simplex* keratitis (HSK) [1].

*Kocuria* spp. are catalase-positive, Gram-positive cocci belonging to the family Micrococcaceae, which comprises 17 species, and are part of the resident flora of the oral cavity and skin. Infectious diseases caused by *Kocuria* spp. include endocarditis, peritonitis, and catheter-related infections [2–4], and affected individuals are often immunocompromised. *Kocuria* spp. are also unusual causative organisms in ocular infectious diseases, and there are a small number of reports on *Kocuria* keratitis [5, 6].

Here, we report the first case of bacterial keratitis caused by *Kocuria* sp. complicated with a shield ulcer in an AKC patient.

## Case presentation

### Case report

A 51-year-old Japanese man presented with a 14-day history of eye pain and visual loss in his left eye. He was referred to our hospital from a local medical eye clinic for comprehensive investigation of the corneal infection due to lack of response after 7 days to medical treatment for HSK (aciclovir ophthalmic ointment once daily and fluorometholone ophthalmic solution three times daily). His medical history included atopic dermatitis for more than 40 years, AKC with a previous corneal shield ulcer, and HSK recurrence in his left eye 5 years previously.

**Fig. 1:**
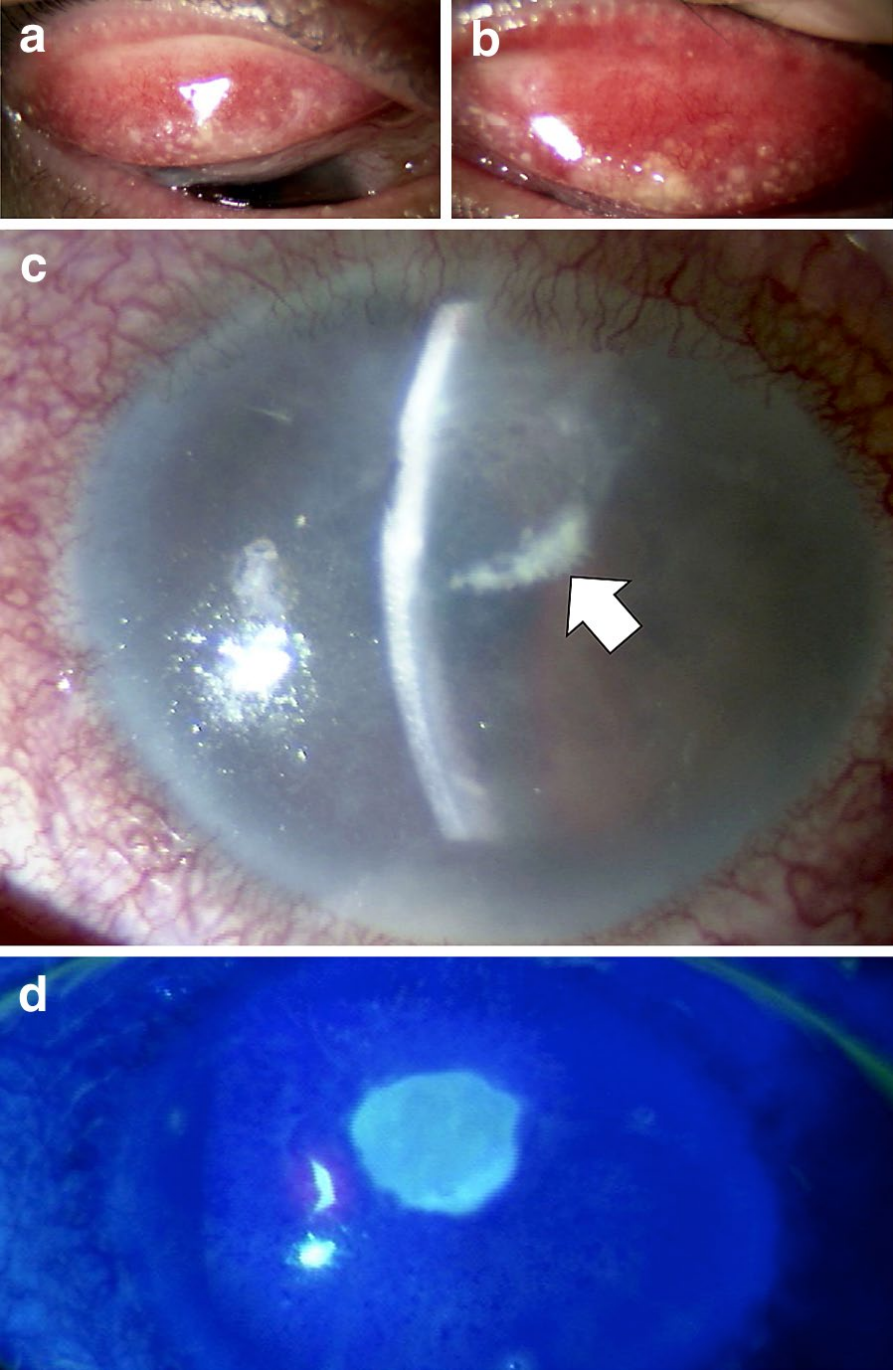
Slit-lamp photographs at the initial clinical evaluation. **a**, **b** Slit-lamp photographs of the upper tarsal conjunctiva of the right (**a**) and left (**b**) eyes. The palpebral conjunctiva have a velvety appearance. **c** A shield ulcer is present in the upper-central cornea. Corneal opacification with a coral-like appearance (*arrow*) is present at 4–6o’clock in the marginal zone of the shield ulcer. **d** The shield ulcer shows positive staining with fluorescein dye

On ocular examination at the initial visit, corrected visual acuity was 20/20 in the right eye and 30 cm hand motion in the left eye. He also exhibited bilateral blepharitis with thickening and hyperpigmentation and Dennie–Morgan folds. The conjunctiva of both eyes had a velvety appearance caused by papillary proliferation, and conjunctival hyperemia and swelling were present in the superior tarsal area (Fig. 1a, b). There was a shield ulcer in the upper-central cornea of the left eye, and the marginal zone of the ulcer had a coral-like appearance due to white stromal opacification (Fig. 1c). The ulcer showed positive staining with fluorescein dye (Fig. 1d). Observation of the anterior chamber was impossible due to corneal opacity; however, abundant keratic precipitates and mild hypopyon were observed.

**Fig. 2:**
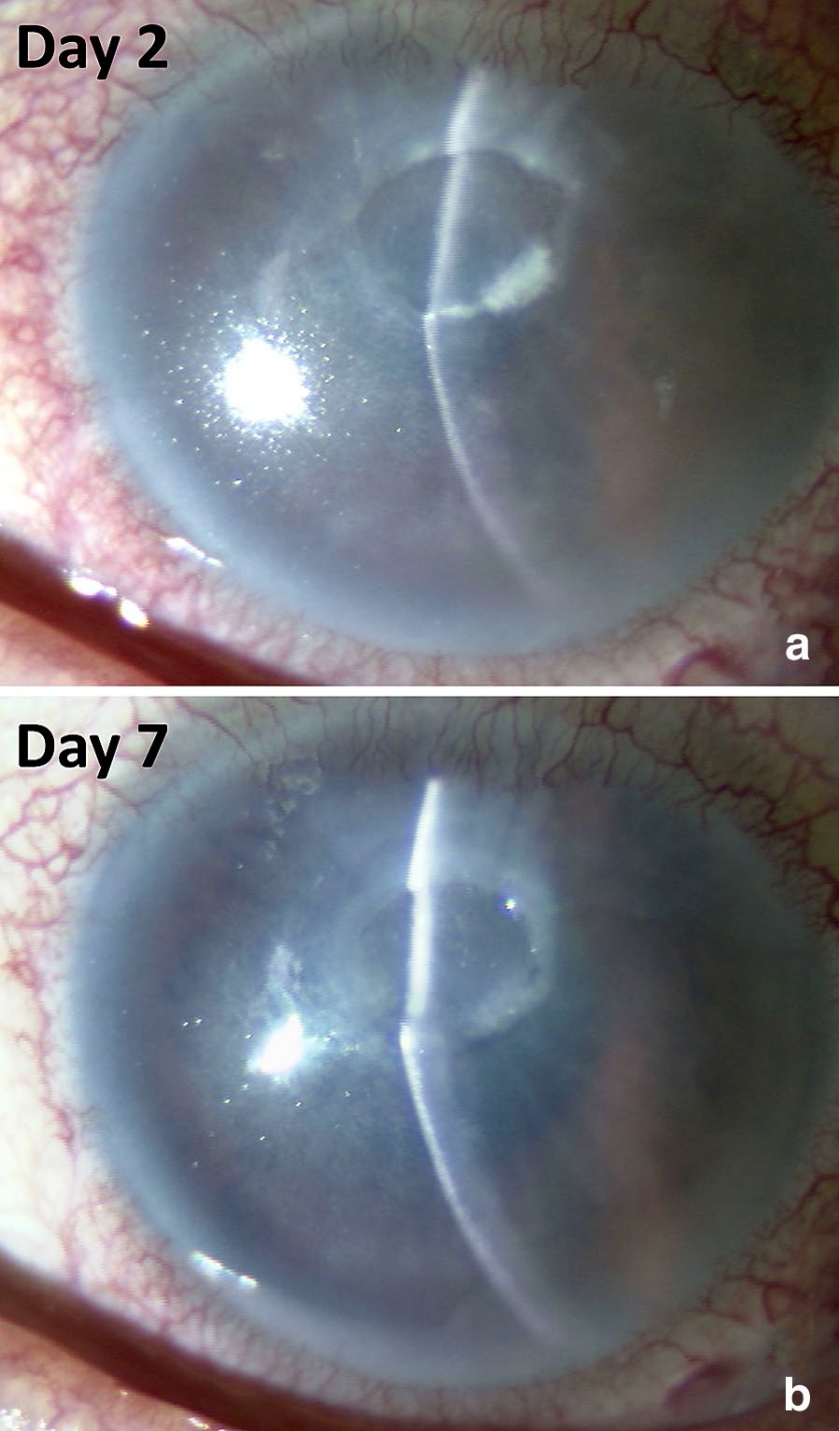
Slit-lamp photographs of the corneal ulcer 2 and 7 days after the initial examination. Corneal opacification with a coral-like appearance shows gradual improvement and has resolved on day 7. **a** Slit-lamp photographs of the corneal ulcer at day 2. **b** Slit-lamp photographs of the corneal ulcer at day7

Corneal scraping was performed to obtain samples for microbiologic examinations, and the patient was started on topical cefmenoxime ophthalmic solution (Bestron^®^ ophthalmic solution 0.5%; Senju Pharmaceutical, Osaka, Japan) administered every 2 hours, sodium cromoglycate ophthalmic solution (Intal^®^ UD ophthalmic solution 2%; Sanofi, Tokyo, Japan) administered four times daily, and ofloxacin ophthalmic ointment (Tarivid^®^ ophthalmic ointment, Santen Pharmaceutical, Osaka, Japan) administered once daily at bed time. With these treatments, corneal stromal opacity and the coral-like appearance gradually improved and resolved within 7 days (Fig. 2a, b).

### Microbiologic examination

The scrapings of the corneal ulcer were inoculated into chocolate agar at the initial visit. In addition, polymerase chain reaction for *Herpes simplex* virus DNA was performed to rule out the possibility of recurrent HSK.

White colonies formed by catalase-positive, Gram-positive cocci growing on the chocolate agar and were further identified as *Kocuria* sp. using matrix-assisted laser desorption/ionization time-of-flight mass spectrometry (MALDI-TOF/MS; Microflex^®^, Bruker Daltonik GmbH, Bremen, Germany). Furthermore, bacterial gene sequence analysis was performed using isolated strain of *Kocuria* sp. from the patient. The 16S rRNA gene sequence identified this strain with *Kocuria koreensis* (Table 1). Homology >98.7% using multiple gene databases (Basic Local Alignment Search Tool (BLAST), https://blast.ncbi.nlm.nih.gov/Blast.cgi) is the criterion for gene sequence identification in our laboratory.

**Table 1:** Results of 16S rRNA gene sequence analysis for the isolated strain of *Kocuria* sp. from the patient

Rank	Name	Pairwise similarity (%)
1	*Kocuria koreensis* strain P31 16S ribosomal RNA gene, partial sequence	789/790 (99%)
2	*Kocuria carniphila* strain CCN 132 16S ribosomal RNA gene, partial sequence	765/795 (96%)
3	*Kocuria rhizophila* strain TA68 16S ribosomal RNA gene, partial sequence	761/791 (96%)
4	*Kocuria salsicia* strain 104 16S ribosomal RNA gene, partial sequence	760/792 (96%)
5	*Kocuria kristinae* strain DSM 20032 16S ribosomal RNA gene, partial sequence	760/792 (96%)
6	*Kocuria halotolerans* strain YIM 90716 16S ribosomal RNA gene, complete sequence	762/795 (96%)
7	*Kocuria marina* strain KMM 3905 16S ribosomal RNA gene, partial sequence	759/792 (96%)
8	*Kocuria gwangalliensis* strain SJ2 16S ribosomal RNA gene, partial sequence	761/795 (96%)
9	*Nesterenkonia lutea* strain YIM 70081 16S ribosomal RNA gene, partial sequence	757/792 (96%)
10	*Kocuria indica* strain NIO-1021 16S ribosomal RNA gene, partial sequence	757/793 (95%)

The results of antimicrobial susceptibility testing for the isolated *Kocuria* sp. are shown in Table 2. The *Kocuria* sp. isolated from the corneal shield ulcer of the AKC patient showed susceptibility to ampicillin, benzylpenicillin, cefaclor, imipenem/cilastatin, and clindamycin. Susceptibility was also shown to vancomycin and levofloxacin, but the minimum inhibitory concentration was 1 μg/mL

The results of polymerase chain reaction for *Herpes simplex* virus DNA were negative in both eyes

**Table 2:** Antimicrobial susceptibility results for the isolated *Kocuria* sp.

Minimum inhibitory concentration (μg/mL)				
ABPC	PCG	CCL	CTX	IPM/CS
≤0.12	≤0.25	≤0.12	1	≤0.06

MEPM	EM	CLDM	VCM	LVFX
0.25	4	≤0.25	1	1

*ABPC* ampicillin, *PCG* benzylpenicillin, *CCL* cefaclor, *CTX* cefotaxime sodium, *IPM/CS* imipenem/cilastatin sodium, *MEPM* meropenem, *EM* erythromycin, *CLND* clindamycin, *VCM* vancomycin, *LVFX* levofloxacin

## Discussion

We report a rare case of *Kocuria koreensis* keratitis complicated with a shield ulcer in an AKC patient. In cases of opportunistic corneal infection complicating AKC, we should consider the possibility of *Kocuria* spp., in addition to *Staphylococcus aureus* and Fungus as causative organisms [7–9], because these species are also considered one of the resident skin flora.

Patients with atopic dermatitis are susceptible to developing skin infections due to alterations in the normal flora, compromised host immunological mechanisms caused by atopic diathesis, and immunosuppression due to administration of certain medications such as steroids and immunosuppressive agents [1, 10]. *Staphylococcus aureus* populations in the resident flora of the skin increase in patients with atopic dermatitis due to reduced antimicrobial peptides, such as human beta-defensin-2 [1, 11, 12]. In chronic AKC patients, *Staphylococcus aureus* colonization of the lid margins may exacerbate the allergic inflammatory reactions in the conjunctiva and cornea, thereby facilitating the development of infectious keratitis. In addition, the use of steroid or other immunosuppressive ophthalmic medications in these patients may predispose them to the development of opportunistic infection. Recognized types of infectious keratitis in AKC patients include staphylococcal keratitis and fungal keratitis [7–9].

The characteristic clinical finding in *Kocuria* keratitis is an echinulate white corneal opacity. Hence, we described the cornea as having a “coral-like appearance” in this case. A case of *Kocuria* keratitis reported by Mattem et al. [5] exhibited corneal opacity with a coral-like appearance, similar to our case. Therefore, this coral-like appearance may be considered a useful diagnostic sign.

In this case, microbiological diagnosis was performed using MALDI-TOF/MS. Identification of *Kocuria* spp. has been performed previously using an automated instrument for identification and antibiotic susceptibility testing (VITEK^®^ 2 system) [13] or 16S ribosomal DNA sequencing [5]. In our diagnostic method for bacterial keratitis, an abrasion sample of infected cornea is applied directly to chocolate agar and cultured, and species of isolated bacteria are identified using MALDI-TOF/MS. Since diagnosis of the bacterial strain and antimicrobial susceptibility testing can be provided simultaneously, these combination of diagnostic method are considered useful for the rapid diagnosis of rare bacterial keratitis cases. Furthermore, The 16S rRNA gene sequence analysis was performed using isolated *Kocria* strain from the patient. The obtained DNA sequence showed 99% homology with *Kocuria koreensis. Kocuria koreensis* is a rare species in causative organism of infectious disease by Kocuria spp. A phylogenetic analysis based on the 16S rRNA gene sequence indicated that *Kocuria koreensis* P31^T^ was most closely related to *Kocuria kristinae* DSM 20031^T^, with 96.9% similarity, and these two strains clustered together in constructed phylogenetic trees [14]. To the best of our knowledge, this is a first report of *Kocuria koreensis* keratitis.

The *Kocuria koreensis* isolated from the corneal shield ulcer in this case showed susceptibility to penicillin and a first-generation cephalosporin antimicrobial agent. Therefore, in this case, the combination therapy of corneal scraping and instillation of the cefmenoxime antibiotic ophthalmic solution was considered effective for *Kocuria* keratitis. However, further investigation on the selection of antibiotics for *Kocuria* spp. isolated from corneal ulcer is needed.

## Conclusions

To the best of our knowledge, this is the first case report of infectious keratitis caused by *Kocuria koreensis* complicating a shield ulcer in an AKC patient. Precise strain diagnosis and accurate determination of the antimicrobial susceptibility to unusual organisms causing bacterial keratitis are important for the effective treatment of keratitis. In conclusion, clinicians should be aware of *Kocuria* keratitis as a corneal complication of AKC, and that rapid diagnosis of *Kocuria* keratitis may improve the visual prognosis.

## Abbreviations

AKC: atopic keratoconjunctivitis
HSK: *Herpes simplex* keratitis
MALDI-TOF/MS: matrix-assisted laser desorption/ionization time-of-flight mass spectrometry
